# Association of betatrophin amounts with 25-(OH)D levels in patients with gestational diabetes mellitus

**DOI:** 10.1097/MD.0000000000025646

**Published:** 2021-04-23

**Authors:** Fuyan Yang, Wenfa Yang, Guohua Wang, Yaqiong Liu, Jun Jin

**Affiliations:** a Obstetrics Department; b Urology Department; c Clinical Lab Department, The Affiliated Lianyungang Hospital of Xuzhou Medical University, Lianyungang, Jiangsu Province, China.

**Keywords:** 25-(OH)D, betatrophin, cord blood, full-term pregnancy, gestational diabetes mellitus, maternal blood

## Abstract

To determine the association of betatrophin amounts with 25-(OH)D levels in gestational diabetes mellitus (GDM) patients, and to provide new targets for the prevention and treatment of GDM.

This study included 40 GDM patients (GDM group) and 37 healthy pregnant women (control group). Betatrophin, 25-(OH)D, fasting blood glucose (FBG), HbA1c, hsCRP, and FINS levels in peripheral blood, as well as betatrophin and 25-(OH)D amounts in cord blood, were measured. Then, associations of betatrophin levels with 25-(OH)D amounts and other indexes were determined.

Maternal (*P* = .011) and cord (*P* = .022) blood betatrophin levels were significantly lower in the GDM group compared with control group. Cord blood betatrophin levels were higher compared with maternal blood amounts in both the GDM and control groups (both *P* = .000). Serum betatrophin levels were positively associated with 25-(OH)D levels (*r* = 0.677, *P* = .000), but negatively associated with hsCRP (*r* = −0.335, *P* = .037) and HOMA-IR (*r* = −0.346, *P* = .031) levels in the GDM group. Fetal weight was higher in the GDM group compared with control group (*P* = .023), and negatively associated with cord blood betatrophin amounts in the GDM group (*r* = −0.342, *P* = .031). However, cord blood betatrophin levels were not significantly associated with body length, Apgar score, and cord blood 25-(OH)D levels in the GDM group (all *P* > .05).

Serum betatrophin and 25-(OH) D levels were positively associated in women with GDM, and both significantly lower compared with control values. Fetal weight was higher in the GDM group and associated with cord blood betatrophin. These findings provide insights into developing new predictive biomarkers or therapeutic targets for GDM.

## 1 Introduction

Gestational diabetes mellitus (GDM), a common complication of pregnancy, refers to any degree of abnormal glucose regulation during pregnancy.^[1]^ Previous studies have shown that the incidence of pregnancy complications is 3- to 6-fold higher in GDM patients compared with control pregnant women. GDM not only increases adverse pregnancy outcomes, including macrosomia, gestational hypertension, stillbirth, and neonatal birth injury, but also augments the risk of metabolic diseases, such as type 2 diabetes mellitus, in both the mothers and newborns. Insulin resistance and reduced secretion of insulin by pancreatic β cells are considered important pathogenic mechanisms of GDM.^[2]^

Betatrophin is a 198 amino acid secreted protein mainly expressed in the liver and adipose tissues. It participates in the metabolism of glucose and lipids, and is closely associated with the development of diabetes and cardiovascular diseases.^[3–6]^ In addition, several studies have reported that betatrophin is closely associated with inflammatory factors, including high-sensitivity C-reactive protein (hs-CRP).^[4]^ Serum betatrophin levels in GDM patients have been shown to differ, to a certain degree, compared with normal pregnant women,^[7]^ suggesting that betatrophin could be associated with the development and progression of GDM. Recent studies have indicated that betatrophin levels in cord blood could reflect intrauterine growth and fetal development.^[8,9]^

Vitamin D is an endocrine hormone, of which 25-(OH)D can pass through the placental barrier. The cord blood levels of vitamin D depend on its maternal amounts. Vitamin D deficiency is associated with several diseases, including type 2 diabetes mellitus and GDM.^[10]^ Previous findings have indicated that 25-(OH)D deficiency is a risk factor for GDM.^[11]^ However, studies investigating the association of betatrophin amounts with 25(OH)D levels in GDM patients are scarce. Hence, this study aimed to investigate betatrophin and 25(OH)D amounts in GDM women and to determine their association, as well as the associations of betatrophin with other clinical indicators. This study could provide insights into the development of new targets for the prevention and treatment of GDM.

## 2 Methods

### 2.1 Subjects

The pregnant full-term GDM women (GDM group) who underwent regular prenatal examinations and delivered through cesarean section at the Affiliated Lianyungang Hospital of Xuzhou Medical University between June 2017 and June 2018 were enrolled in this study. GDM was diagnosed based on the National Health and Family Planning Commission in 2011 guidelines.^[12]^ Briefly, a 75-g oral glucose tolerance test (OGTT) was performed at 24 to 28 weeks of gestation. The cut-off value of fasting glucose, and 1- and 2-hours venous blood glucose levels were 5.1 mmol/L, 10.0 mmol/L, and 8.5 mmol/L, respectively. A diagnosis of GDM was reached with glucose amounts equal to or exceeding the respective cut-off values.

The GDM group received standard management based on clinical guidelines. No drugs, such as insulin, were administered to help control blood glucose levels. In addition, consecutive healthy pregnant women who delivered by cesarean section during the same period were included in the control group. Inclusion criteria were:

1) women aged 19 to 40 years;
2) educational level of junior middle school or higher;
3) no psychological issues and the ability to communicate well, and
4) willingness to participate in this study.

Subjects in both groups had no previous history of diabetes mellitus, acute inflammatory diseases, gastrointestinal disease, liver disease, renal disease, fetal deformity, and history of smoking and/or drinking.

This study was approved by the Ethics Committee of the Affiliated Lianyungang Hospital of Xuzhou Medical University. All participants provided signed informed consent prior to enrollment.

### 2.2 Biographical and medical data collection

Age, gestational week, medical history, and laboratory parameters were recorded for all study participants. Height and body weight were measured to determine the body mass index (BMI) in kg/m^2^. The Apgar score was obtained with the newborn kept warm and cleared respiratory tract. The newborn's weight and length were measured with an electronic scale and a regular tape measure, respectively. The Apgar score in newborns incorporates the heart rate, respiration, muscle tone, reflex irritability, and skin color, with a subscore of 0, 1, or 2 per factor; the total Apgar score was obtained by adding all subscores.^[13]^

### 2.3 Sample collection

Upon reaching full-term, 5 mL of fasting blood was obtained from the cubital vein on the day of the cesarean section. In addition, 5 mL of cord blood was collected from the proximal end after delivery of the fetus but before the delivery of the placenta. Maternal and cord blood samples were centrifuged (Xiangyi, China) at 3000 rpm, and the serum was collected and stored at −80°C until use.

### 2.4 Measurement methods

Betatrophin levels were measured with the USCNK ELISA kit (Wuhan, China) as directed by the manufacturer, and a full-wavelength microplate reader (SpectraMax Plus 384, MD) was used for absorbance reading. The Cobas instrument (Roche) was used to measure fasting insulin (FINS; 20162404356, Roche Diagnostic GmbH) and 25-(OH)D (20142405153, Roche Diagnostic GmbH) levels by electrochemiluminescence (ECLIA). Then, 75 g OGTT and fasting blood glucose (FBG) level assessment employed the hexokinase method on an AU5821 biochemical analyzer (Beckman Coulter). Hemoglobin A1c (HbA1c) was measured by HPLC (VARIANT-II, Bio-Rad) on a D-10 hemoglobin A1c analyzer (Bio-Rad). To determine whether the inflammatory factor hs-CRP could influence betatrophin levels, immuno-turbidimetric assay was performed on an immage-800 immunoassay system (Beckman Coulter) to measure hs-CRP levels. Homeostasis model assessment (HOMA) was used to calculate insulin resistance index (HOMA-IR) by the following equation: HOMA-IR = FINS × FBG/22.5.

### 2.5 Statistical analysis

The SPSS17.0 software (SPSS Inc., Chicago) was used for data processing and analysis. Quantitative data were presented as mean ± standard division (mean ± SD). Student's *t* test was performed for comparing normally distributed data, while non-normally distributed data were assessed by the Wilcoxon test. Pearson correlation analysis was performed to assess associations between indicator pairs. *P* < .05 was considered statistically significant.

## 3 Results

### 3.1 General characteristics and biochemical indicators

Forty pregnant GDM patients and 37 healthy pregnant women were included in this study. Age (*P* = .101), gestational week (*P* = .275), and BMI (*P* = .066) before pregnancy were not significantly different between the 2 groups of pregnant women. However, BMI increase (BMI) during pregnancy was significantly different between the 2 groups (*P* = .030). Maternal blood 25-(OH)D levels were significantly lower in the GDM group compared with the control group (*P* = .000). In addition, 75 g OGTT-FBG, 1-hour blood glucose, 2-hour blood glucose, hsCRP, FINS, and HOMA-IR were all significantly higher in the GDM group compared with the control group (all *P* = .000; Table 1).

**Table 1:** General clinical data and biochemical indicators in the 2 study groups.

Factors	GDM group (n = 40)	Control group (n = 37)	*t*	*P*
Age (yr)	30.41 ± 3.91	28.83 ± 4.32	1.663	.101
Gestational age (wk)	38.66 ± 1.01	38.92 ± 1.05	−1.099	.275
BMI before pregnancy (kg/m^2^)	23.77 ± 2.78	22.48 ± 3.24	1.864	.066
ΔBMI (kg/m^2^)	5.45 ± 1.66	6.48 ± 2.36	−2.207	.030
75 g OGTT (mmol/L)				
FBG	5.07 ± 0.50	4.25 ± 0.40	7.905	.000
1st hour	10.10 ± 0.74	7.68 ± 0.82	13.612	.000
2nd hour	8.39 ± 0.51	6.51 ± 0.70	13.542	.000
Maternal blood 25-(OH)D (ng/mL)	17.44 ± 7.06	23.22 ± 4.69	−4.228	.000
hsCRP (mg/mL)	6.44 ± 3.08	3.64 ± 1.90	4.780	.000
FINS (mU/L)	18.87 ± 7.79	12.64 ± 4.48	4.242	.000
HOMA-IR	4.24 ± 1.89	2.37 ± 0.87	5.555	.000
HbA1C (%)	5.39 ± 0.52	4.89 ± 0.41	4.561	.000
FBG (mmol/L)	5.04 ± 0.64	4.20 ± 0.52	6.255	.000

### 3.2 Betatrophin levels

Betatrophin levels in maternal blood were significantly lower in the GDM group compared with the control group (*P* = .011). Betatrophin levels in cord blood were also significantly lower in the GDM group compared with control values (*P* = .022). In addition, betatrophin levels in cord blood were significantly higher compared with maternal blood amounts in both the GDM and control groups (both *P* = .000; Table 2).

**Table 2:** Betatrophin levels in the GDM and control groups.

	Maternal blood (ng/mL)	Cord blood (ng/mL)	*t*	*P*
GDM group	0.61 ± 0.30	1.14 ± 0.39	−6.768	.000
Control group	0.79 ± 0.31	1.36 ± 0.45	−6.286	.000
*t*	2.609	2.341		
*P*	.011	.022		

### 3.3 Associations of betatrophin and 25-(OH)D amounts with other indicators

Maternal betatrophin levels were positively associated with maternal 25-(OH)D levels in the GDM group (*r* = 0.677, *P* = .000), and negatively associated with hsCRP (*r* = −0.335, *P* = .037) and HOMA-IR (*r* = −0.346, *P* = .031) levels. However, maternal betatrophin levels were not associated with gestational week, age, BMI before pregnancy, ΔBMI, HbA1C, FINS, and FBG (all *P* > .05). Maternal 25-(OH)D levels were positively associated with gestational week (*r* = 0.445, *P* = .005) and hsCRP (*r* = 0.436, *P* = .006) levels in maternal blood, and negatively associated with HbA1C (*r* = −0.365, *P* = .022) and HOMA-IR (*r* = −0.430, *P* = .006) levels. There were no significant associations with age, BMI before pregnancy, ΔBMI, FINS, and FBG (all *P* > .05; Table 3).

**Table 3:** Associations of betatrophin, 25-(OH)D, and clinical biochemical indicators in the GDM group.

	Maternal blood betatrophin		Maternal blood 25-(OH)D	
Factor	*r*	*P*	*r*	*P*
Gestational week	0.250	.117	0.445	.005
Age	−0.008	.959	0.012	.943
ΔBMI	−0.153	.353	−0.186	.258
BMI before pregnancy	−0.023	.891	0.014	−.933
FINS	0.172	.294	0.046	.781
HbA1C	−0.323	.245	−0.365	.022
FBG	−0.129	.434	−0.081	.622
hsCRP	−0.335	.037	0.436	.006
HOMA-IR	−0.346	.031	−0.430	.006
Maternal blood 25-(OH)D	0.677	.000		

### 3.4 Fetal outcomes and associations with cord blood betatrophin levels in the GDM group

Fetal outcomes were assessed between the GDM and control groups (Table 4). Except for fetal weight that showed a significant difference between the GDM and control groups (3708.75 ± 341.87 g vs 3525.95 ± 349.07 g, *P* = .023), all other parameters, including newborn body length and Apgar scores at 1 and 5 minutes were comparable in both groups (all *P* > .05).

**Table 4:** Fetal outcomes in both study groups.

Factor	GDM group (n = 40)	Control group (n = 37)	*t*	*P*
Fetal weight (g)	3708.75 ± 341.87	3525.95 ± 349.07	−2.321	.023
Body length (cm)	50.65 ± 1.72	50.46 ± 1.52	−0.514	.609
Apgar score				
1 min (score)	8.93 ± 0.27	8.95 ± 0.23	0.368	.714
5 min (score)	9.93 ± 0.27	9.97 ± 0.16	0.941	.350

Betatrophin and 25-(OH)D levels in cord blood were 1.16 ± 0.43 ng/mL and 15.0736 ± 6.77 ng/mL, respectively, with no significant correlation between them (*r* = 0.081, *P* = .850), as summarized in Table 5. Further correlation analysis indicated that fetal weight was significantly correlated with cord blood betatrophin amounts in the GDM group (*r* = −0.342, *P* = .031) in the GDM group, while newborn body length and Apgar scores at 1 and 5 minutes were not (all *P* > .05).

**Table 5:** Associations of cord blood betatrophin levels with fetal outcomes in the GDM group.

Factor	*r*	*P*
Fetal weight (g)	−0.342	.031
Body length (cm)	−0.023	.889
Apgar score		
1 min (score)	0.129	.426
5 min (score)	0.076	.642
Cord blood 25-(OH)D	0.081	.850

## 4 Discussion

In this study, the levels of betatrophin, 25-(OH)D, and related clinical biochemical indicators in GDM patients were measured, and the associations of these indicators were then determined. To the best of our knowledge, this is the first study determining the association of betatrophin amounts with 25-(OH)D levels in GDM women. Our results also suggested that cord blood betatrophin levels might be associated with the overtly higher fetal weight in GDM. The present findings provide further insights into the metabolic dysfunctions observed in GDM patients.

Previous findings revealed that betatrophin regulates glucose and lipid metabolism.^[14]^ Abnormal betatrophin secretion has been associated with the development of metabolic diseases, as well as various abnormal health conditions, including obesity, type 2 diabetes mellitus,^[15,16]^ and GDM.^[17]^ A study also reported that plasma betatrophin levels in pregnant women are reduced with increasing gestational weeks but returned to normal levels after delivery.^[18]^ However, there is still ambiguity as to the levels of betatrophin between GDM and normal pregnant women. A meta-analysis^[19]^ and a study by Ebert et al^[20]^ demonstrated that plasma betatrophin levels are higher in GDM patients compared with normal healthy pregnant women. In contrast, Huang et al^[21]^ reported that betatrophin levels are lower in pregnant GDM women compared with normal healthy pregnant women. Inconsistencies in these findings may be due to several reasons. Previous studies have indicated that ELISA kits from distinct vendors could yield different results for betatrophin levels. Indeed, C- or N-terminal ELISA reagents for betatrophin measurement purchased from distinct companies could have different affinities, therefore suggesting inconsistent measurement levels.^[22,23]^ Secondly, Vitamin D status could result in inconsistent betatrophin measurement levels.^[24]^ Thirdly, pregnancy duration could influence betatrophin levels.^[18]^

In this study, we found that serum betatrophin levels were significantly lower in women with GDM compared with normal healthy pregnant women (*P* = .011). Insulin resistance is a pathological process essential for the development of GDM. Correlation analysis in this study demonstrated that betatrophin levels were negatively associated with HOMA-IR. This suggested that lower serum betatrophin levels in GDM patients could be caused by insulin resistance. In addition, we showed that betatrophin levels in cord blood were significantly higher compared to levels in maternal blood (both *P* = .000), in both the GDM and control groups. This suggested that betatrophin was secreted during the fetal stage, and could play a role in fetal growth and development. A previous study performed by Wawrusiewicz-Kurylonek et al^[25]^ corroborated the present findings, indicating that intrauterine hyperglycemia could increase placental betatrophin secretion. Pearson correlation analysis performed in this study revealed a negative correlation between maternal blood betatrophin levels and hsCRP (*r* = −0.335, *P* = .037). However, several previous clinical studies reported a positive association between betatrophin levels and hsCRP.^[26]^ Although our findings are inconsistent with previous findings, they suggest that inflammation could be involved in the regulation of betatrophin levels.

25-(OH)D is the major storage form of vitamin D in the human body. It has a relatively long half-life (3 weeks) and is not influenced by parathyroid hormone and calcium levels. Hence, it is considered the best indicator of vitamin D status.^[27]^ Several studies have shown that 25-(OH)D participates in various pathophysiological processes, including inflammatory responses, immunity, and the metabolism of glucose and lipids.^[28]^ In this study, we found that maternal blood 25-(OH)D levels were positively associated with hsCRP (*r* = 0.436, *P* = .006) in GDM group. Adequate vitamin D levels not only regulate calcium influx and consequently increase insulin secretion in pancreatic β cells, but also control proliferation and apoptosis in pancreatic β cells to promote insulin secretion.^[29]^ Zhou et al^[30]^ reported that vitamin D deficiency could lead to abnormal insulin secretion to increase the risk of insulin resistance. A study revealed that low vitamin D levels are associated with GDM, and serum 25-(OH)D levels are significantly lower in GDM patients compared with healthy pregnant women with normal glucose tolerance.^[30]^ We found that 25-(OH)D levels in maternal blood were significantly lower in the GDM group compared with healthy pregnant women, and showed a positive association with gestational weeks. This suggested that during pregnancy, the requirement for vitamin D increases gradually, and vitamin D deficiency could be associated with GDM development. These findings were consistent with Liao et al.^[2]^ Maternal 25(OH)D is transferred to the fetus through the umbilical cord, and hence maternal vitamin D is the source of fetal vitamin D. We demonstrated that 25-(OH)D levels in cord blood were not significantly associated with betatrophin levels (*r* = 0.081, *P* = .850). 25-(OH)D levels in cord blood are influenced by 25-(OH)D levels in maternal blood, while betatrophin secretion by the fetus is regulated by the fetus itself, and not by amniotic fluid and maternal betatrophin levels.^[31]^

A recent study described betatrophin as a novel target gene of the vitamin D receptor in human hepatocytes, which is induced by vitamin D and bile acid.^[32]^ Another report suggested that 25-(OH)D is an independent factor affecting betatrophin levels, and is positively associated with betatrophin levels.^[33]^ We found that serum levels of 25-(OH)D and betatrophin were positively associated in GDM patients (*r* = 0.677, *P* = .000). This suggested an additional mechanism by which 25-(OH)D affects pancreatic β cells.

Multiple reports have assessed the association of GDM with offspring overweight, with discrepant results.^[34,35]^ Of the fetal outcomes evaluated, only fetal weight was overtly higher in the GDM group compared with control patients. We also showed that cord blood betatrophin levels might be associated with the overtly higher fetal weight in GDM, which has not been reported yet.

There were several limitations in this study. First, the samples were derived from only one time point (full-term pregnancy) and may not reflect changes in indicator levels during pregnancy. Secondly, 25-(OH)D amounts are affected by various factors, including ethnicity, solar radiation, diet, and vitamin D supplementation. This could influence the present findings. Thirdly, this was a cross-sectional study with a small sample cohort. Any causal relationships between the 2 factors could not be identified. Additional multi-center studies assessing larger cohorts from different ethnicities are needed to validate the current findings.

In summary, serum betatrophin and 25-(OH) levels were positively associated in pregnant women with GDM. Our results also demonstrated that cord blood betatrophin might be negatively correlated with the overtly higher fetal weight in GDM. These parameters could be used as valuable biomarkers and therapeutic targets in GDM.

## Acknowledgments

The authors would like to thank all study participants enrolled in this study.

## Author contributions

Fuyan Yang performed the study and wrote the manuscript. Wenfa Yang performed the statistical analysis and wrote the manuscript. Guohua Wang supervised the study and revised the manuscript. Yaqiong Liu performed the statistical analysis. Jun Jin performed laboratory test measurements.

**Conceptualization:** Fuyan Yang.

**Data curation:** Wenfa Yang, Yaqiong Liu.

**Formal analysis:** Wenfa Yang, Yaqiong Liu.

**Methodology:** Jun Jin.

**Supervision:** Guohua Wang.

**Writing – original draft:** Fuyan Yang.

**Writing – review & editing:** Fuyan Yang, Wenfa Yang, Guohua Wang, Yaqiong Liu, Jun Jin.
